# Limited antibody specificity compromises epitranscriptomic analyses

**DOI:** 10.1038/s41467-019-13684-3

**Published:** 2019-12-11

**Authors:** Mark Helm, Frank Lyko, Yuri Motorin

**Affiliations:** 1 Institute of Pharmacy and Biochemistry, Johannes Gutenberg-University Mainz, Staudingerweg 5, 55128 Mainz, Germany; 2 Division of Epigenetics, DKFZ-ZMBH Alliance, German Cancer Research Center, Heidelberg, Germany; 3 IMoPA, UMR7365 CNRS-University of Lorraine, Biopôle, 9 Avenue de la Forêt de Haye, 54505 Vandœuvre-lès-Nancy, France

**Keywords:** RNA, RNA modification

## Abstract

A controversial discussion on the occurrence of the RNA modification m^1^A in mRNA takes a new turn, as an antibody with a central role in modification mapping was shown to also bind mRNA cap structures.

## Antibodies as tools for modification mapping

RNA modifications are chemical alterations that diversify the functionality of the canonical RNA building blocks. New mapping methods have focused considerable attention on the modification content of eukaryotic mRNA and its potential for the regulation of gene expression.

A recent study reports on the differential specificity of antibodies directed against the RNA modification m^1^A, and how they impact interpretation of the resulting modification maps^1^. Particularly striking is the revelation that a previously used antibody binds cap structures in addition to m^1^A, and that previously reported m^1^A mapping results likely contain an abundance of false positives at the 5′-end of mRNA.

While variabilities in antibody specificity are commonplace, the study by Jaffrey and colleagues will perhaps drive home the importance of specificity validation for antibody-dependent RNA modification mapping. What makes this case so relevant? Antibody biotechnology—and the many important tools it has produced—often rely on a combinatorial approach to identifying molecules with high affinity to specific epitopes. In the context of peptide binding, the variable region of typical antibodies recognizes an epitope of 5–12 amino acids^2^, which present a significant diversity of functional groups to mediate affinity and specificity. This situation is different for nucleic acids, as they possess comparatively limited structural diversity, and correspondingly less well-defined primary epitopes.

Antibodies directed against nucleic acid modifications have played an important role in the fields of epigenetics and epitranscriptomics. Enrichment of DNA containing 5mC by methylated DNA immunoprecipiatation (MeDIP) has been a widely used technique in epigenetics for decades, before its RNA version, MeRIP became popular. Indeed, MeRIP experiments have been published as early as the 1980s^3^, albeit not under that acronym. Only the combination with RNA-Seq transformed it into a breakthrough technology for the RNA modification field in 2012, when two teams independently reported maps of m^6^A modifications in mammalian mRNA^4,5^. Since then, several antibodies have been used for mapping various RNA modifications^6^ with substantial impact for the community. Problems with antibody specificity have been discussed^7^, but have remained largely under-recognized.

## Off-target binding of antibodies is a widespread problem

More recently, studies in the DNA modification field have begun to identify sources of artifacts. For example, issues of inherent cross-reactivity can be amplified by low abundance of the primary epitope. This is exemplified by the cross-reactivity of antibodies to contaminating bacterial nucleic acids that can confound the modification analysis of eukaryotic DNA^8^. Of note, the existence of dm^6^A and dm^4^C in DNA of higher eukaryotes^9,10^ has been questioned by an antibody-independent analysis^11^. Another recent study has shown that several antibodies directed toward DNA modification cross-react with short tandem repeats in a modification-independent manner, which can in turn generate experimental noise as high as 99%^12^.

Given that the development of such antibodies includes a conjugation step to a protein via the oxidized sugar moiety of a modified nucleoside^13^, modification-specific antibodies could be expected to recognize the modified nucleobase irrespective of whether they are found in DNA or RNA. Thus, the demonstration of specificity problems in MeRIP experiments in the current publication by Grozhik et al. should not come as a surprise; rather it is a long awaited, experimentally thorough and convincing demonstration of antibody-dependent artifacts in the RNA modification field^1^. In addition to providing experimental guidelines for the field as a whole, the study also uncovers the unexpected binding of a commercially available anti-m^1^A antibody to cap structures. Furthermore, the study provides important clarifications in the controversial discussion regarding the number of m^1^A residues present in mammalian mRNA, which have been reported in several publications^14–17^. More specifically, the results reported by Grozhik et al. suggest that m^1^A is infrequent in mRNA, and that the prevalence of this modification was substantially overestimated in previous studies. A comparative assessment of two m^1^A antibodies led to vastly different results in MeRIP-type experiments, likely pointing to a general problem in the field. For one, specifications and specificity claims for a given antibody should be taken with caution and preferably confirmed for each application using the relevant controls. Secondly, it should now be clear that confirming antibody specificity by simple methods such as dot blot experiments should be considered insufficient^18^. Of the many validation techniques that the field has developed^6^, Grozhik et al. judiciously applied mass spectrometry and thin layer chromatography to characterize the physicochemical properties of material isolated by MeRIP^1^. A systematic characterization of the various antibodies commonly used might be highly beneficial, as was shown in the not-so-distant field of histone modifications. There, a systematic evaluation of antibody specificity was conducted using peptide-arrays, and revealed substantial specificity problems already several years ago^19^.

## Considerations beyond antibody specificity

On a more fundamental level, one might question if a single methyl group in a nucleic acid fragment can really provide a sufficient level of selectivity for MeRIP or other similar techniques. Although our understanding of binding modes is limited, it is clear that the primary epitope can not only be the modification itself (i.e. a methyl group), but can extend to the modified nucleobase (i.e. adenine) and beyond. It follows that all adenines present in the RNA also compete for binding, albeit with lower affinity than the methylated adenine. In such a situation, the enrichment will be governed by the relative affinities toward modified and unmodified residues, and by their relative abundances. This, in turn, means, that any adenine in unmodified RNA (including polyA-tails) may give rise to non-specific binding, especially if the modification is of low abundance. It is thus perhaps not surprising that enrichment factors reported in MeRIP experiments are as low as 4–10-fold^20^. For relatively abundant modifications, such as m^6^A, this may still be sufficient to produce credible mapping results. However, this may not be the case for less abundant RNA modifications.

With respect to MeRIP in general, several additional problems exist that extend beyond antibody specificity, and which could skew the results of modification mapping experiments. A number of these problems are related to the experimental design of Illumina sequencing and library preparation protocols used. For example, early modification calling reports have neglected the use of unique molecular identifiers (UMI)^6^, leading to artificial amplification of noise by PCR^21^. Potential artifacts resulting from RNA-Seq adapter design have also been discussed^22^. Moreover, problems in the computational analysis of RNA-Seq data, such as ambiguity in read mapping, are among the known error sources^23^. Finally, the field needs standards for stringent statistical data analysis^24^. Taking into account all these limitations, the massive datasets obtained by newly reported mapping techniques for RNA modification analysis should be considered collections of candidate modification sites, rather than experimentally confirmed modification landscapes. Therefore, further efforts should aim to develop and apply multiple orthogonal methods for the validation of modification sites and genome-wide patterns^6^.
